# Toward Precision Therapeutics in Pulmonary Arterial Hypertension

**DOI:** 10.1016/j.jacbts.2026.101681

**Published:** 2026-08-18

**Authors:** Jason Hong

**Affiliations:** Division of Pulmonary and Critical Care Medicine, Department of Medicine, University of California, Los Angeles, Los Angeles, California, USA

**Keywords:** biopsy, endothelial dysfunction, network medicine, precision medicine, pulmonary arterial hypertension

**Figure:**
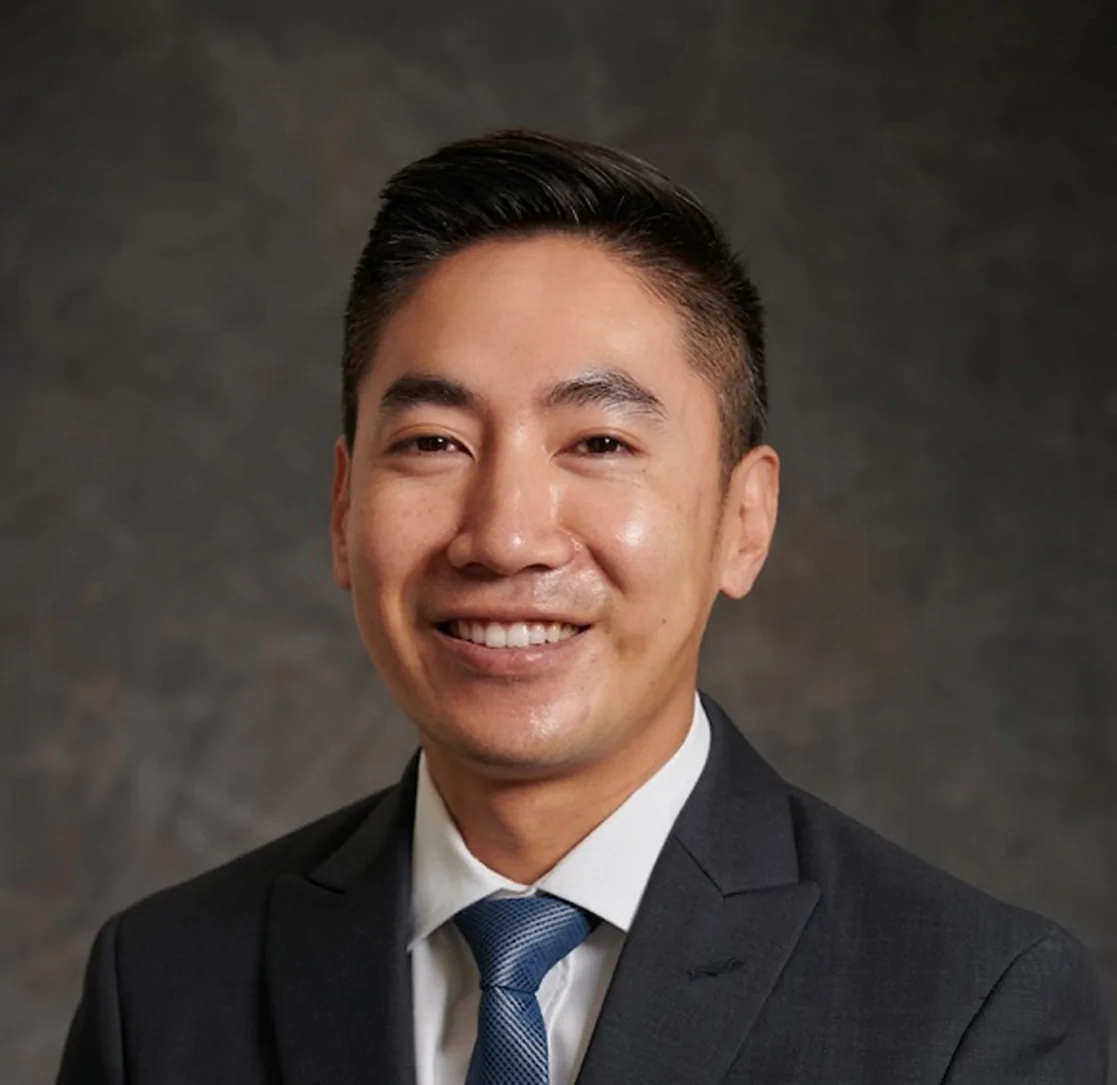

Pulmonary arterial hypertension (PAH) has entered an era of unprecedented therapeutic opportunity. Yet despite an expanding therapeutic armamentarium, clinicians remain limited in their ability to determine which treatment is most likely to benefit an individual patient. Treatment decisions are still largely based on clinical phenotype and risk stratification, although patient-specific biology has yet to inform therapeutic selection. As our understanding of the molecular heterogeneity of PAH has deepened, an increasingly important challenge is translating these biological insights into individualized therapeutic decisions.

In this issue of *JACC: Basic to Translational Science*, Wang et al^1^ take an important step toward addressing this challenge by integrating pulmonary artery endothelial cell biopsies obtained during routine right heart catheterization with network medicine and systems pharmacology to construct individualized interactomes. Rather than using transcriptomic data simply to characterize disease biology, the investigators ask whether patient-specific molecular networks can help explain differences in therapeutic response. Greater alignment between individualized interactomes and the molecular targets of prescribed PAH therapies was associated with favorable changes in natriuretic peptide levels and Registry to Evaluate Early and Long-Term PAH Disease Management (REVEAL) risk score. These findings provide an intriguing proof-of-concept that patient-specific vascular biology may ultimately inform therapeutic selection.

The significance of this work extends beyond its analytical methodology. Precision medicine in PAH has traditionally focused on characterizing molecular heterogeneity through increasingly sophisticated approaches, including genomic, transcriptomic, proteomic, and biomarker analyses.^2^^,^^3^ These approaches have expanded our understanding of disease heterogeneity and identified biologically distinct endophenotypes.^4^ Previous applications of network medicine have primarily focused on identifying disease modules, prioritizing therapeutic targets, and facilitating drug repurposing.^5^^,^^6^ Wang et al^1^ build on these advances by applying individualized interactomes to an inherently clinical question—whether alignment between a patient's molecular network and the targets of prescribed therapies is associated with clinical response. In doing so, this study begins to bridge the long-standing gap between molecular characterization and therapeutic decision-making.

Another notable aspect of this work is its use of pulmonary artery endothelial cell biopsies obtained during routine clinical care. Lung tissue has historically been inaccessible except at transplantation or autopsy, limiting efforts to interrogate patient-specific pulmonary vascular biology. The ability to obtain pulmonary artery endothelial cells during clinically indicated right heart catheterization^7^^,^^8^ provides a practical window into one of the principal cell types implicated in PAH pathogenesis. When coupled with individualized network analyses, this approach has the potential to transform a routinely performed diagnostic procedure into a platform for biological phenotyping.

Nevertheless, several important challenges remain before this approach can be translated into routine clinical practice. Foremost, this study demonstrates an association between patient-specific interactomes and therapeutic response but does not establish their clinical utility for guiding therapeutic selection. Whether individualized interactomes can improve clinical outcomes when used to guide therapy remains an open question that will require prospective validation. In addition, the current workflow—including endothelial cell isolation, ex vivo expansion, RNA sequencing, interactome reconstruction, and systems pharmacology—is resource-intensive and will require further streamlining before widespread clinical implementation becomes feasible. Finally, although endothelial dysfunction is a defining feature of PAH, the disease is fundamentally multicellular, involving dynamic interactions among endothelial cells, smooth muscle cells, fibroblasts, pericytes, and immune populations. Future precision medicine frameworks will likely benefit from integrating these diverse cellular interactions, together with right ventricular biology, into increasingly comprehensive patient-specific models.

Importantly, these limitations should be viewed in the context of what this study represents: an early proof-of-concept rather than a finished clinical platform. Many technologies that are now routinely used in clinical medicine initially required specialized technical infrastructure before ultimately becoming clinically accessible. This approach may follow a similar trajectory, particularly as advances in single-cell transcriptomics, spatial biology, and computational methods continue to accelerate.

Ultimately, precision medicine fulfills its promise only when biological insight changes clinical decision-making. This work offers a vision of how patient-specific biology could eventually be translated into therapeutic decision-making. Whether this approach ultimately reshapes the management of PAH remains to be determined, but it represents an important step toward a future in which therapy is guided not only by disease severity, but also by the unique biology of each patient.

## Funding Support and Author Disclosures

The author has reported that he has no relationships relevant to the contents of this paper to disclose.
